# Sex and Gender Influence on Cardiovascular Health in Sub-Saharan Africa: Findings from Ghana, Gambia, Mali, Guinea, and Botswana

**DOI:** 10.5334/gh.1146

**Published:** 2022-09-01

**Authors:** Rubee Dev, Divine Favour-Ofili, Valeria Raparelli, Hassan Behlouli, Zahra Azizi, Karolina Kublickiene, Alexandra Kautzky-Willer, Maria Trinidad Herrero, Louise Pilote, Colleen M. Norris, on behalf of the GOING-FWD Consortium

**Affiliations:** 1 Faculty of Applied Science, School of Nursing, University of British Columbia, Vancouver, Canada; 2 Department of Epidemiology, Biostatistics and Occupational Health, School of Population and Global Health, McGill University, Montreal, Quebec, Canada; 3 Faculty of Nursing, University of Alberta, Edmonton, Alberta, Canada; 4 Department of Translational Medicine, University of Ferrara, Ferrara, Italy; 5 University Center for Studies on Gender Medicine, University of Ferrara, Ferrara, Italy; 6 Research Institute of McGill University Health Centre, Division of Clinical Epidemiology McGill University, Montreal, Quebec, Canada; 7 Centre for Outcomes Research and Evaluation, McGill University Health Centre Research Institute, Montreal, QC, Canada; 8 Department of Clinical intervention, Science and Technology (CLINTEC), Section for Renal Medicine, Karolinska Institute and Karolinska University hospital, Stockholm, Sweden; 9 Department of Internal Medicine III, Division of Endocrinology and Metabolism, Gender Medicine Unit, Medical University of Vienna, Vienna, Austria; 10 Clinical & Experimental Neuroscience (NiCE-IMIB-IUIE), School of Medicine. University of Murcia, Murcia, Spain; 11 Faculty of Medicine and Dentistry and School of Public Health, University of Alberta, Edmonton, Alberta, Canada

**Keywords:** Cardiovascular health, Cardiovascular diseases, sub-Saharan Africa, Sex, Gender

## Abstract

**Background:**

There is an upsurge of cardiovascular diseases (CVDs) in sub-Saharan Africa (SSA). Irrespective of biological sex, gender-related factors could be the precursor of these conditions.

**Objective:**

To examine the associations between biological sex, gender-related variables, and cardiovascular health (CVH) risk factors in SSA countries.

**Methods:**

We used data from the STEPwise approach to surveillance of risk factors for non-communicable disease survey, conducted in adults from Ghana, Gambia, Mali, Guinea, and Botswana. The main outcome was CVH, measured through the health index with values ranging from 0 (worst) to 5 (best or ideal) CVH. Multivariable logistic regression was applied to determine the gender-related factors related to poorer CVH (index less than 4).

**Results:**

Data included 15,356 adults (61.4% females, mean age 36.9 years). The prevalence of hypertension (21.6% vs. 13.8%) and overweight/obesity (48.3% vs. 27.5%) was higher among females as compared to males. Females were more likely to be unemployed (17.3% vs. 9.7%) or reported unpaid work (36.8% vs. 15.2%). Overall, females showed worse CVH than males (OR_female_ = 0.95, 95% CI:0.91–0.99). Being married was associated with better CVH compared with being single, more so for males (OR_male_ = 1.09, 95% CI:0.96–1.24, p_interaction_ < 0.01). Males with unpaid work (OR_male_ = 1.28, 95% CI:1.12–1.47) had better CVH than their unpaid female counterparts (OR_female_ = 1.08, 95% CI:1.01–1.17).

**Conclusion:**

In SSA populations, being female was associated with poorer CVH given the disproportionate burden of hypertension and overweight/obesity. Gender-related factors such as marital status and unpaid work were associated with better CVH in males compared to females.

## Introduction

The 21^st^ century marked the onset of an epidemiological transition in Africa, with a surge of noncommunicable diseases (NCDs) taking the lead as the primary cause of death [1]. With the contemporary double burden of communicable and non-communicable diseases, cardiovascular diseases (CVDs) are rising in sub-Saharan Africa (SSA) [2,3], predominantly driven by increased rates of hypertension, smoking, and obesity [4]. In SSA, CVDs are the most frequent causes of NCDs deaths, responsible for approximately 13% of all deaths and 37% of all NCDs deaths [2]. Between 1990 and 2013, SSA remained the only region of the world where CVD related mortality increased [3]. Of concern, the burden of CVD is expected to double by 2030 [3]. The significant increase in the number of deaths from CVDs, namely hypertensive heart disease, stroke, and heart failure, raises a possibility of an emerging epidemic in SSA that is preventable [5]. Moreover, an upsurge of the CVDs poses an additional burden on an already over-burdened healthcare systems [6] that needs to be addressed.

Earlier studies have established sex differences in the risk and occurrence of CVDs [7,8]; however, they have mostly ignored the intersectionality of sex, gender, and other demographic characteristics. A review of the evidence for Ghana, Nigeria, South Africa, Sudan, and Tanzania indicated a poor health system response to the increasing risk of CVDs with no discussion on the risk associated with sex and gender [9]. Whilst sex-based studies, especially in NCDs, are gaining more advocacy globally, the sex and gender research gap and data paucity is highly visible in the African context. While sex differences in cardiovascular risk and events have been investigated, their association with sociocultural gender has not received the same attention. Unfortunately, many studies use the terms gender and sex interchangeably. Furthermore, studies with sex-stratified analysis that lack considerations of the influences of gender may not present a complete description of cardiovascular health/events. This directly impacts the design and implementation of interventions designed to meet the targets for Sustainable Development Goal 3 and achieve a 30% reduction in premature mortality due to NCDs [10]. Therefore, this study aimed to investigate and describe the associations between biological sex, gender-related variables, and cardiovascular health risk factors in SSA countries.

## Methods

### Study design

This is a cross-sectional study using deidentified data from the population-wide, sub-national/national World Health Organization’s (WHO)-STEPwise Approach to surveillance of risk factors for non-communicable disease (STEPS) survey data [11]. The design of the STEPS survey has been published elsewhere [11]. Briefly, the survey included Step I: administration of a questionnaire to elicit demographic information and lifestyle behaviors; Step II: physical measurements (height, weight, waist/hip circumference, blood pressure, and heart rate); and Step III: biochemical assessments (fasting blood glucose, total cholesterol, and urine sample for testing of sodium, potassium, and creatinine levels) of respondents. The measurements were carried out at the home of the survey participants immediately after the conclusion of Step I, and assessments were carried out the next day at a common place for all the participants. The surveys were conducted between 2006–2014. The inclusion criteria consisted of participants of working age (18–69 years). For the purposes of this analysis, we selected STEPS data from five SSA countries (Botswana, Gambia, Ghana, Guinea, and Mali) for which data were available in a Microdata repository.

### Explanatory variables

Self-reported biological sex and gender-related variables (i.e., education, occupation/employment, marital status, household income, and household size) were identified using the GOING-FWD methodology [12]. Because of country-specific differences in data coding or collection, data harmonization and recategorization was done for the identified gender-related variables. Biological sex in this study was defined as binary, male or female; education level was categorized into three groups: less than secondary, secondary, and post-secondary; marital status was categorized into three groups: single/never married, widow/separated/divorced, and married/common in law; work status was categorized into three groups: paid, unpaid, and unemployed; and household size was categorized into five groups: 1, 2, 3, 4, and ≥5 people in the household. Age of the respondents was recorded as a continuous variable in the dataset and was categorized into six groups: <20, 20–29, 30–39, 40–49, 50–59, and 60–69 years.

### Outcome variable (STEPS-HEART index)

Cardiovascular health (CVH) derived from the STEPS-HEART index was the main outcome variable in this study. Variables measured in the survey (i.e., smoking, hypertension, diabetes, obesity/overweight, and daily consumption of fruits and vegetables) were used to construct a composite labeled the STEPS-HEART index; value ranging from 0 to 5 [13]. The STEPS-HEART index is an adaptation of the previously published CANHEART health index tool [14]. It provides a composite quantitative measure (of 0 [worst] to 5 [best]) of CVH factors— smoking, diabetes mellitus, elevated blood pressure, obesity/overweight, and daily consumption of fruits and vegetables [14]. Performance of ideal CVH behavior within each factor was positively coded with a score of 1, while non-ideal performances were scored 0. Each metric of ideal cardiovascular health is defined in Appendix, Table 1. These definitions were collectively adapted from the American Heart Association, World Health Organization, and/or Center of Disease Control and Prevention definitions or recommendations. Later, a binary variable of STEPS-HEART index (value of 4 and 5—better CVH coded as 1, and value less than 4—poorer CVH coded as 0) was formed for the purpose of analysis.

**Table 1:** Descriptive characteristics of survey respondents, by biological sex (n = 15356).


	N^1^	OVERALL	BIOLOGICAL SEX	

			FEMALE (N = 9425)	MALE (N = 5931)
		
		N (%) OR MEAN [SD]	N (%) OR MEAN [SD]	N (%) OR MEAN [SD]

**Demographic variables**				

**Country/Survey year**	15356			

Ghana, 2006		2662 (17.3)	1773 (18.8)	889 (15.0)

Gambia, 2010		4103 (26.7)	2335 (24.8)	1768 (29.8)

Mali, 2007		2401 (15.6)	1441 (15.3)	960 (16.2)

Guinea, 2009		2288 (14.9)	1230 (13.1)	1058 (17.8)

Botswana, 2014		3902 (25.4)	2646 (28.1)	1256 (21.2)

**Age (in years)**	15356	37.5 [12.5]	36.9 [12.3]	38.5 [12.8]

**Age categories**	15356			

<20		917 (6.0)	568 (6.0)	349 (5.9)

20–29		4789 (31.2)	3138 (33.3)	1651 (27.8)

30–39		4121 (26.8)	2559 (27.2)	1562 (26.3)

40–49		2770 (18.0)	1069 (17.1)	1161 (19.6)

50–59		1962 (12.8)	1101 (11.7)	861 (14.5)

60–69		797 (5.2)	459 (4.8)	347 (5.9)

**Gender variables**				

**Education level**	15295			

Less than secondary		8350 (54.6)	5548 (59.1)	2802 (47.4)

Secondary		5514 (36.1)	3195 (34.0)	2319 (39.3)

Post-secondary		1431 (9.4)	645 (6.9)	786 (13.3)

**Marital status**	15278			

Single/Never married		4575 (29.9)	2486 (26.5)	2089 (35.4)

Married/Common in law		9484 (62.1)	5888 (62.8)	3596 (61.0)

Widow/Separated/Divorced		1219 (8.0)	1006 (10.7)	213 (3.6)

**Work status**	14559			

Paid		8363 (57.4)	4051 (45.9)	4312 (75.2)

Unpaid		4114 (28.3)	3244 (36.8)	870 (15.2)

Unemployed		2082 (14.3)	1527 (17.3)	555 (9.7)

**Household size**	14955			

1 person		2530 (16.9)	1512 (16.5)	1018 (17.6)

2 persons		4224 (28.2)	2667 (29.1)	1557 (26.8)

3 persons		2733 (18.3)	1776 (19.4)	957 (16.5)

4 persons		1623 (10.9)	963 (10.5)	660 (11.4)

≥5 persons		3845 (25.7)	2238 (24.4)	1607 (27.7)

**Annual household income (USD)**	11819			

<20000		9106 (77.0)	5312 (75.5)	3794 (79.4)

≥20000		2713 (23.0)	56 (24.5)	67 (20.6)

**Cardiovascular risk-factors**				

**Smoking**	15179			

Smoker		1859 (12.2)	210 (2.2)	1649 (28.4)

Non-smoker		13320 (87.8)	9169 (97.8)	4151 (71.6)

**Overweight/obesity**	14673			

BMI < 25		8767 (59.7)	4642 (51.7)	4125 (72.5)

BMI ≥ 25		5906 (40.3)	4343 (48.3)	1563 (27.5)

**Leisure physical activity**	1576			

<30 mins activity		226 (14.3)	138 (20.0)	88 (9.9)

≥30 mins activity		1350 (85.7)	552 (80.0)	798 (90.1)

**Fruit and vegetable consumption**	10700			

<5 servings/day		7205 (67.3)	4490 (68.1)	2715 (66.2)

≥5 servings/day		3495 (32.7)	2107 (31.9)	1388 (33.8)

**Hypertension**	11907			

Yes		2247 (18.9)	1671 (21.6)	576 (13.8)

No		9660 (81.1)	6057 (78.4)	3603 (86.2)

**Diabetes**	6807			

Yes		311 (4.6)	215 (4.9)	96 (4.0)

No		6496 (95.4)	4162 (95.1)	2334 (96.0)

**Outcome variable**				

**STEPS-HEART Index (Africa)**	4271			

0		4 (0.1)	2 (0.1)	2 (0.1)

1		80 (1.9)	26 (1.8)	54 (1.9)

2		415 (9.7)	109 (7.4)	306 (10.9)

3		1464 (34.3)	441 (29.9)	1023 (36.6)

4		1696 (39.7)	605 (41.0)	1091 (39.0)

5		612 (14.3)	291 (19.7)	321 (11.5)


*Note*: BMI (Body Mass Index); SD (Standard Deviation). ^1^ Number of complete observations (excludes missing data). STEPS-HEART index value of ≥4 indicates better CVH and value <4 indicates poor CVH.

### Statistical Analysis

Means, standard deviation, frequencies and percentages were used to describe the study population. Multivariate logistic regression models were used to investigate the association between CVH and gender-related factors in the African context. Sex-stratified logistic regression models were fitted to identify the impact of the association between the various gender variables and the CVH within each sex stratum. Age was included in all the models. Two-way interaction between the sex and gender-related factors were tested by including an interaction term in bivariate models. We also conducted p for trend test to look at a potential trend between age range and household size across CVH categories. Missing data were imputed using the multiple imputation method. All the statistical analyses were executed using SAS software version 9.4 (SAS Institute, Cary, North Carolina).

### Data imputation

A complete case analysis when data are not missing completely at random is inefficient and can potentially lead to biased results [15]; hence, we employed multiple imputation to account for the missing data. Missing values were imputed using SAS, which employs a fully conditional specification (FCS) algorithm [16]. The FCS method is an iterative Markov Chain Monte Carlo procedure that sequentially imputes missing values for all covariates included starting from the first variable with missing values by specifying a linear regression or logistic regression model for each continuous or categorical variable, respectively. We used 30 imputations with 20 (by default) iterations and included work status (6% missing), household size (3% missing), and annual household income (23% missing) variables for the imputation.

## Results

### Descriptive characteristics of respondents

Descriptive characteristics of the study population stratified by biological sex are presented in Table 1. Among 15,356 respondents, 61.4% were females (mean age 36.9 years). A quarter of the respondents were from Gambia (26.7%) and Botswana (25.4%) each. Female respondents were more likely to be married (62.8% vs. 61.0%), to have lower formal education (59.1% vs. 47.4%), and report less paid work (45.9% vs. 75.2%) compared to their male counterparts.

**Figure 1:**
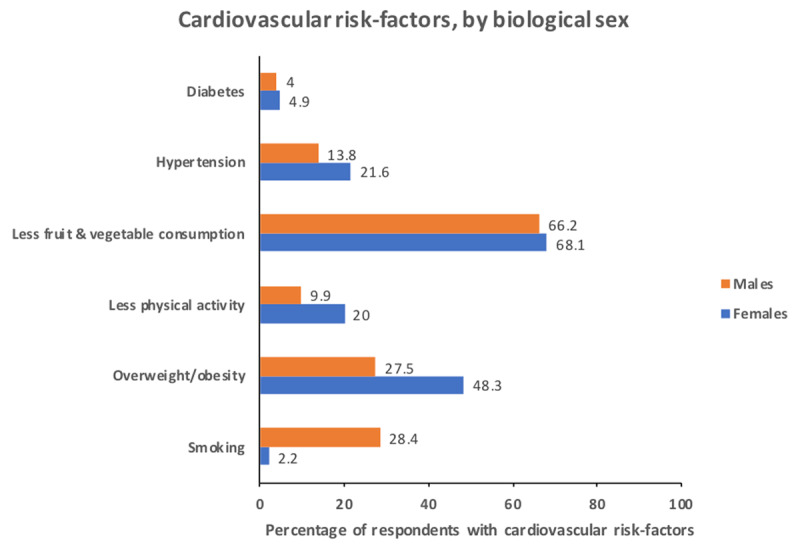
Cardiovascular risk-factors of the respondents by biological sex.

Over a third of the study population had three or more cardiovascular risk factors with a higher prevalence in females than males (Figure 1). Although close to 80% of the study population was less than 50 years of age, the prevalence of hypertension was high, especially in women (with a 21.6% reported prevalence). Diabetes prevalence was similar between sexes but higher than reported population prevalence for these countries, likely related to the very high prevalence of overweight/obesity, especially among women (48.3%). Smoking prevalence was very high in males, almost a third (28.4%), while it remained low in females (2.2%). Of the persons reporting engagement in leisure physical activities, a majority (85.7%) reported meeting the ideal daily physical activity, however, in the wider population, access to fruits and vegetables was low in both sexes (31.9% and 33.8%); only a third had access to at least 5 servings per day (Table 1).

### Association of gender-related factors with CVH by sex

The respondents had a mean CVH value of 3.5 and a median value of 4, indicating intermediate to good cardiovascular health across the study population. Overall, females showed poorer CVH compared to males (OR_female_ = 0.95, 95% CI: 0.91–0.99). CVH decreased with increasing age for both sexes. Females in the oldest age group (OR_female_ = 0.37, 95% CI: 0.26–0.53) had poorer CVH compared to their male counterparts (OR_male_ = 0.67, 95% CI: 0.47–0.97, p_interaction_ < 0.01). While unpaid workers showed better CVH (OR_female_ = 1.08, 95% CI: 1.01–1.17 and OR_male_ = 1.28, 95% CI: 1.12–1.47), those who were unemployed (OR_female_ = 0.87, 95% CI: 0.79–0.95 and OR_male_ = 0.78, 95% CI: 0.68–0.89) showed poorer CVH for both males and females. Females with household size of ≥5 showed better CVH compared to males (OR_female_ = 1.39, 95% CI: 1.24–1.56 vs. OR_male_ = 1.18, 95% CI: 1.05–1.34, p_interaction_ < 0.05) (Table 2).

**Table 2:** Multiple imputation logistic regression model for assessing association of gender-related variables with cardiovascular health, overall and by biological sex.


CARDIOVASCULAR HEALTH (STEPS-HEART INDEX)	OVERALL		FEMALE		MALE		P INTERACTION
		
	OR (95% CI)	P-VALUE	OR (95% CI)	P-VALUE	OR (95% CI)	P-VALUE	

**Sex (Female)**	0.95 (0.91–0.99)	0.01					

**Age**							

<20	2.06 (0.50–8.48)	0.32	2.35 (0.56–9.81)	0.25	1.54 (0.36–6.55)	0.56	0.15

20–29 (ref)	–	–	–	–	–	–	–

30–39	1.14 (0.85–1.51)	0.39	1.25 (0.93–1.68)	0.16	1.07 (0.79–1.44)	0.68	<0.05

40–49	0.76 (0.56–1.02)	0.07	0.77 (0.57–1.04)	0.09	0.79 (0.57–1.08)	0.14	0.60

50–59	0.65 (0.49–0.87)	0.01	0.58 (0.43–0.78)	<0.001	0.80 (0.58–1.11)	0.18	<0.01

60–69	0.48 (0.35–0.66)	<0.001	0.37 (0.26–0.53)	<0.001	0.67 (0.47–0.97)	<0.05	<0.001

**Education level**							

Less than secondary (ref)	–		–		–		

Secondary	0.99 (0.94–1.05)	0.81	0.93 (0.86–1.02)	0.11	1.10 (1.01–1.20)	<0.05	<0.01

Post-secondary	0.91 (0.84–0.99)	<0.05	0.87 (0.77–0.98)	<0.05	0.92 (0.83–1.03)	0.16	0.54

**Marital status**							

Single/Never married (ref)	–		–		–		

Married/Common in law	1.11 (1.04–1.18)	<0.01	1.05 (0.98–1.14)	0.16	1.09 (0.96–1.24)	0.19	<0.01

Widow/Separated/Divorced	0.99 (0.89–1.09)	0.79	1.03 (0.93–1.15)	0.57	0.93 (0.75–1.16)	0.54	0.59

**Work status**							

Paid (ref)	–		–		–		

Unpaid	1.14 (1.08–1.21)	<0.001	1.08 (1.01–1.17)	<0.05	1.28 (1.12–1.47)	<0.001	0.23

Unemployed	0.84 (0.79–0.90)	<0.001	0.87 (0.79–0.95)	<0.01	0.78 (0.68–0.89)	<0.001	0.60

**Household size**							

1 (ref)	–		–		–		

2	0.93 (0.86–0.99)	<0.05	0.88 (0.81–0.96)	<0.01	0.99 (0.89–1.11)	0.97	0.79

3	0.96 (0.88–1.05)	0.36	0.96 (0.87–1.07)	0.47	0.95 (0.84–1.09)	0.49	0.29

4	0.99 (0.90–1.09)	0.92	0.99 (0.88–1.13)	0.98	0.98 (0.84–1.15)	0.83	0.93

≥5	1.30 (1.19–1.43)	<0.001	1.39 (1.24–1.56)	<0.001	1.18 (1.05–1.34)	<0.01	<0.05


*Note*: OR (Odds Ratio); CI (Confidence Interval); STEPS-HEART index (4 or 5: Better CVH vs. <4: Poorer CVH).

### Association of gender-related factors with CVH by country

Table 3 demonstrates the country specific CVH of the respondents. Females showed poorer CVH in all countries except in Gambia, where the association was in reverse (OR = 1.07, 95% CI: 0.97–1.19); however, the finding is not statistically significant. CVH decreased with increasing age in all five countries (p for trend < 0.001). Respondents with post-secondary education as compared to less than secondary education in Gambia showed significantly poorer CVH (OR = 0.67, 95% CI: 0.53–0.83). Respondents who were married or common in law had better CVH compared to single or never married individuals, mainly in Gambia (OR = 1.12, 95% CI: 0.97–1.28) and Mali (OR = 1.19, 95% CI: 0.99–1.43). Unpaid respondents compared to their paid counterparts had better CVH except in Guinea, where unpaid respondents had poorer CVH (OR = 0.91, 95% CI: 0.72–1.15). CVH seemed to be better with increasing number of household members in all countries (p for trend < 0.001). There was no evidence of heterogeneity in the associations between sexes (p_interaction_ > 0.05), except for age and education level in Gambia.

**Table 3:** Multiple imputation logistic regression model for assessing association of gender-related variables with cardiovascular health, by country.


CARDIOVASCULAR HEALTH (STEPS-HEART INDEX)	GHANA		GAMBIA		MALI		GUINEA		BOTSWANA	
				
	OR (95% CI)	P-VALUE	OR (95% CI)	P-VALUE	OR (95% CI)	P-VALUE	OR (95% CI)	P-VALUE	OR (95% CI)	P-VALUE

**Sex (Female)**	0.73 (0.66–0.80)	<0.001	1.07 (0.97–1.19)	0.15	0.98 (0.88–1.09)	0.67	0.99 (0.89–1.11)	0.94	1.00 (0.93–1.09)	0.93

**Age**										

<20	NA		NA		2.76 (1.87–4.06)	<0.001	1.57 (0.17–14.18)	0.69	1.34 (0.15–11.52)	0.79

20–29 (ref)	–	–	–	–	–	–	–	–	–	–

30–39	1.28 (1.09–1.49)	<0.01	1.38 (1.19–1.59)	<0.001	1.04 (0.82–1.32)	0.76	1.18 (0.72–1.95)	0.51	1.14 (0.72–1.81)	0.57

40–49	0.90 (0.76–1.06)	0.21	0.88 (0.74–1.03)	0.11	0.61 (0.48–0.77)	<0.001	0.83 (0.51–1.36)	0.47	0.97 (0.61–1.55)	0.91

50–59	0.87 (0.72–1.04)	0.12	0.77 (0.63–0.93)	<0.01	0.72 (0.55–0.94)	<0.05	0.68 (0.41–1.13)	0.14	0.59 (0.37–0.95)	<0.05

60–69	0.56 (0.41–0.75)	<0.001	0.59 (0.42–0.82)	<0.01	0.50 (0.34–0.73)	<0.001	0.51 (0.28–0.91)	<0.05	0.57 (0.34–0.98)	<0.05

**Education level**										

Less than secondary (ref)	–		–		–		–		–	

Secondary	0.89 (0.79–1.01)	0.07	1.16 (0.99–1.37)	0.06	0.96 (0.69–1.35)	0.82	0.89 (0.73–1.08)	0.24	0.99 (0.89–1.11)	0.90

Post-secondary	1.07 (0.89–1.29)	0.46	0.67 (0.53–0.83)	<0.001	0.84 (0.45–1.57)	0.59	1.08 (0.84–1.39)	0.54	1.03 (0.90–1.17)	0.66

**Marital status**										

Single/Never married (ref)	–		–		–		–		–	

Married/Common in law	0.99 (0.89–1.13)	0.93	1.12 (0.97–1.28)	0.11	1.19 (0.99–1.43)	0.06	0.99 (0.84–1.19)	0.98	0.95 (0.82–1.11)	0.53

Widow/Separate/Divorce	0.95 (0.79–1.13)	0.56	0.89 (0.70–1.12)	0.32	0.88 (0.63–1.22)	0.42	0.75 (0.56–1.01)	0.06	0.91 (0.72–1.15)	0.44

**Work status**										

Paid (ref)	–		–		–		–		–	

Unpaid	1.03 (0.79–1.34)	0.85	1.19 (1.02–1.39)	<0.05	1.19 (0.97–1.48)	0.09	0.91 (0.72–1.15)	0.44	1.14 (1.01–1.29)	<0.05

Unemployed	0.98 (0.77–1.23)	0.84	0.80 (0.63–1.03)	0.07	0.87 (0.71–1.07)	0.19	1.11 (0.76–1.61)	0.58	0.99 (0.89–1.11)	0.92

**Household size**										

1 (ref)	–		–		–		–		–	

2	0.93 (0.81–1.08)	0.35	1.03 (0.90–1.17)	0.66	0.97 (0.72–1.31)	0.86	0.99 (0.81–1.21)	0.94	0.95 (0.82–1.09)	0.44

3	0.93 (0.78–1.09)	0.36	1.02 (0.89–1.16)	0.80	0.92 (0.66–1.29)	0.64	1.02 (0.82–1.27)	0.85	1.02 (0.86–1.21)	0.81

4	0.99 (0.81–1.23)	0.97	1.03 (0.86–1.22)	0.77	0.99 (0.69–1.43)	0.99	0.98 (0.76–1.28)	0.93	0.95 (0.76–1.18)	0.62

≥5	1.02 (0.84–1.24)	0.84	1.25 (1.09–1.42)	<0.01	1.16 (0.95–1.42)	0.15	1.17 (0.96–1.44)	0.12	1.17 (0.94–1.47)	0.14


*Note*: NA (Not available); OR (Odds Ratio); CI (Confidence Interval); STEPS-HEART index (4 or 5: Better CVH vs. <4: Poorer CVH).

## Discussion

In this SSA population cohort, the cardiovascular risk factor prevalence was high especially in females who exhibited high levels of hypertension (21.6%) and almost half of whom were overweight and/or obese (48.3%). The cumulative cardiovascular risk factor level was higher in females than males, and this association was compounded by deleterious gender-related factors, mostly socioeconomic, which put females at a greater disadvantage compared to males. In the SSA countries surveyed, females reported worse CVH than males in all countries.

High prevalence of hypertension among females in our study cohort is in contrast with the studies that show men having higher blood pressure regardless of their race and ethnicity across all age groups [17]. The high prevalence of hypertension among the females in SSA population could be related to the high prevalence of overweight/obesity. Studies conducted in SSA countries have shown existing sex disparities in overweight/obesity where the increase in prevalence was accounted for almost entirely by females [18,19]. Furthermore, the relationship between obesity and hypertension is well established [20,21]. The rising prevalence of overweight/obesity in this young population of females is worrisome as it is associated with increasing risks of CVDs, particularly hypertension and diabetes that are known risk factors of myocardial infarction, heart failure, stroke, and cognitive decline [21]. In order to impact the rising epidemic of hypertension in women in SSA, gender factors should be considered in the interventions aimed at the prevention and or control of hypertension. Gendered lifestyle factors, such as low consumption of fruits and vegetables and limited physical activity, that are predominant risk factors for women [22], should be taken into account to address the epidemic of CVD risk.

Marital status is an important gendered factor known to predict a range of health outcomes, including CVD. This study identified that marital status was associated with better CVH in both sexes, marriage being more beneficial for males than females, and mainly in Gambia and Mali. In general, men derive more health benefits from marriage than women [23]. Our results are in contrast to a study conducted in Ghana that reported significantly higher odds of hypertension among women who were married while no association was found in men [24]. Marital status was found to be an independent risk factor for CVD in different SSA countries for both women and men [24,25]. Country-specific targeted public health interventions and policies, including more effective risk factor assessment and awareness programs in community settings, keeping in mind the impact of marital status, should be considered in attempting to improve CVH.

Males who reported doing unpaid work reported better CVH compared to their unpaid female counterparts. Women typically spend disproportionately more time on unpaid care work than men. This is often in addition to their paid work, thus creating the “double burden” of work for women [26]. Africa operates a predominantly patriarchal system, with household chores mainly the responsibility of women, in contrast to men, who are the primary earners. With globalization and increased advocacy for education, women now have to manage household chores as well as external labor [27]. The unequal distribution of unpaid work between women and men not only restricts them from participating in paid employment opportunities but also appears to impact their CVH [28]. Addressing issues concerning gendered differences in care responsibilities through policy may impact better gender equality but also result in improved health for both men and women.

Larger household size in our study conferred a better cardiovascular risk profile. Dependent on the age of household members, CVH may improve with larger households. Adults more than 18 years of age may economically contribute to the household income, which in turn results in improved health. Higher income is related to better health conditions, while lower income means more exposure to health risk factors [29]. Higher income families may have better access to a nutritious diet as well as more opportunities for physical activity, whereas families with lower income may be confronted with more stressful situations, leading to reduced health behaviors, which are detrimental to health [30]. Family income, which may be a proxy for larger household size, impacts health and should be further investigated as an important gender-related factor.

### Strengths and limitations

This study has several strengths. The data were collected from a nationally representative survey of the African population that included data on a range of lifestyle and cardiovascular risk-factors, making it possible to examine the gender-related factors associated with CVH. Also, our study contributes much needed evidence to the scarcely investigated relationships between sex-gender intersectionality and CVH, with an echo to the need for further research in this area. There are, however, some limitations that need to be considered. First, data used in this study are over 10 years old, but they are the most recent available data for each specific country, and the prevalence of cardiac risk factors may only be increasing due to the economic transition that is currently happening in SSA. Second, by design, only those who were up to 69 years of age were recruited in the survey. Due to the exclusion of the older population, it is likely that the prevalence of respondents with metrics of less than 4 is underestimated; however, this should not affect the associations between gender-related factors and CVH observed. Third, while creating a STEPS-HEART index, we were not able to include a physical activity variable from CVD risk-factors due to the high proportion of missing data. Fourth, more than a quarter of the study sample is represented from a country with the least population i.e., Gambia, which could limit the representativeness and therefore generalizability of study findings. Finally, we were restricted to the number of gender-related factors that were measured in the survey.

## Conclusions

This study highlights an alarmingly high prevalence of CVD risk factors, mainly overweight/obesity and hypertension among females in SSA population. Socioeconomic gender-related factors were found to contribute to the CVH of both sexes. Being married and doing unpaid work was more predictive of better CVD health in men compared to women. With the rising prevalence of CVDs in SSA, it will be important to consider the gender-related factors while implementing preventive programs and creating effective health policies. Future research should be directed in exploring the association of other important gender-related factors, such as child/adult care responsibilities and number of hours spent on care taking activities, which could impact CVH of an individual.

## Data Accessibility Statements

The WHO-STEPS data are publicly accessible from the Central Data Catalog of NCD Microdata Repository (https://extranet.who.int/ncdsmicrodata/index.php/catalog/central).
